# Visual Exploration While Walking With and Without Visual Cues in Parkinson’s Disease: Freezer Versus Non-Freezer

**DOI:** 10.1177/15459683231201149

**Published:** 2023-09-29

**Authors:** Lisa Graham, Jordan Armitage, Rodrigo Vitorio, Julia Das, Gill Barry, Alan Godfrey, Claire McDonald, Richard Walker, Martina Mancini, Rosie Morris, Samuel Stuart

**Affiliations:** 1 Department of Sport, Exercise and Rehabilitation, Northumbria University, Newcastle, UK; 2 Northumbria Healthcare NHS Foundation Trust, North Shields, UK; 3 Department of Computer and Information Science, Northumbria University, Newcastle, UK; 4 Gateshead Health NHS Foundation Trust, Gateshead, UK; 5 Department of Neurology, Oregon Health & Science University, Portland, OR, USA

**Keywords:** Parkinson’s disease, freezing of gait, eye-tracking, visual exploration, saccades, fixations, walking, visual cues

## Abstract

**Background:**

Visual cues can improve gait in Parkinson’s disease (PD), including those experiencing freezing of gait (FOG). However, responses are variable and underpinning mechanisms remain unclear. Visuo-cognitive processing (measured through visual exploration) has been implicated in cue response, but this has not been comprehensively examined.

**Objective:**

To examine visual exploration and gait with and without visual cues in PD who do and do not self-report FOG, and healthy controls (HC).

**Methods:**

17 HC, 21 PD without FOG, and 22 PD with FOG walked with and without visual cues, under single and dual-task conditions. Visual exploration (ie, saccade frequency, duration, peak velocity, amplitude, and fixation duration) was measured via mobile eye-tracking and gait (ie, gait speed, stride length, foot strike angle, stride time, and stride time variability) with inertial sensors.

**Results:**

PD had impaired gait compared to HC, and dual-tasking made gait variables worse across groups (all *P* < .01). Visual cues improved stride length, foot strike angle, and stride time in all groups (*P* < .01). Visual cueing also increased saccade frequency, but reduced saccade peak velocity and amplitude in all groups (*P* < .01). Gait improvement related to changes in visual exploration with visual cues in PD but not HC, with relationships dependent on group (FOG vs non-FOG) and task (single vs dual).

**Conclusion:**

Visual cues improved visual exploration and gait outcomes in HC and PD, with similar responses in freezers and non-freezers. Freezer and non-freezer specific associations between cue-related changes in visual exploration and gait indicate different underlying visuo-cognitive processing within these subgroups for cue response.

## Introduction

Freezing of gait (FOG) is a common motor symptom affecting people with Parkinson’s Disease (PwPD),^1^ which commonly leads to falls.^2^ FOG is defined as short, episodic periods of reduced forward motion of the feet, despite the intention to walk.^3^ FOG often occurs during situations like gait initiation, turning, and dual-task walking,^4,5^ with occurrence increasing with disease progression.^6^ Falls can result in injury and contribute to increased fear of falling, reduced activity levels, poor quality of life, and caregiver stress.^7^ Thus highlighting the importance of, and need for, strategies and therapies to reduce falls and fall risk factors (ie, FOG).^8^ Pharmacological treatments such as dopaminergic medication have been shown to reduce the duration and occurrence of FOG.^9^ Despite this, evidence for the use of medication for FOG is limited and inconclusive,^10^ with many patients displaying no improvement.^11^ Implementation of non-pharmacological intervention for FOG, such as cueing has been shown to reduce falls and improve mobility in PwPD.^12^ Visual cues (normally a transverse line to step over) are thought to be the most effective strategy for people with FOG, as they are more visually dependent for effective gait than those without FOG.^13^ This is due to impairment in their proprioceptive feedback from the lower limbs.^14,15^ Visual cues provide spatial information used for the initiation or ongoing facilitation of gait,^16^ which helps those with FOG to overcome gait deficits. Specifically, visual cueing can enhance various gait characteristics such as step length^17-19^ and improve gait initiation.^20^ However, the underlying mechanisms for cue response remain poorly understood.^21^ This has led to variation in intervention responses^18^ and selective short-term improvements in selective gait outcomes (ie, only step length or reduced variability).^22^

Underlying and integrated visual and cognitive (termed visuo-cognitive) mechanisms have been implicated in visual cue response in PwPD.^23-25^ Indeed, our recent research has shown that cortical brain regions involved in visuo-cognitive processing (ie, parietal and occipital cortices) are involved in visual cue response in those with FOG compared to those without FOG.^26^ Visual cues may help to focus visuo-cognitive processing on specific task relevant regions of interest (eg, the lines to step over^27,28^), as real-world environments are increasingly visually complex and those with FOG struggle to inhibit visual distractors (ie, they look at thing unrelated to the task they are doing). One method of measuring visuo-cognitive processing in real-time is by recording saccadic (fast, jerk-like) eye movements,^29^ as eye movements are a proxy measurement for visuo-cognition. Saccades are the movements between eye fixations, and form the basis for visual exploration and are influenced by visuo-cognitive neural processes,^30^ subsequently, providing a means to explore the mechanisms behind visual cue responses. The frequency of saccades (visual exploration) is reduced in PwPD during walking compared to healthy older adults, which has been linked to deficits in visuo-cognitive, particularly attentional, processes.^31,32^ However, research has illustrated that visual cueing can ameliorate deficits and improve both saccade frequency (ie, the number of saccades per second)^25^ and fixation number (ie, number of pauses of areas of interest)^19,33^ when walking in PwPD, which suggests that visual cueing may drive visual exploration and be underpinned by visuo-cognitive (particularly attentional) processes. However, to date there have been no studies that have investigated comprehensive visual exploration (both saccadic and fixation outcomes) and its response to visual cueing in individuals with and without FOG.^34^ The lack of comprehensive outcomes likely relates to the poor sampling frequency (ie, 30-50 Hz) of previous eye-trackers that only allow for saccadic or fixational counts to be obtained accurately.^35^ Advances in eye-tracking technology now allows for comprehensive eye-tracking outcomes to be provided at high resolution (ie, >100 Hz) during mobile tasks, such as walking. Comprehensive visual exploration outcomes will allow for greater understanding of visuo-cognitive processes underpinning cue response, as well as exploration of whether improvements in visual exploration are related to gait improvements in PwPD,^23^ which should allow tailoring of future visual cue interventions.^23^

This study aims to: (1) examine comprehensive visual exploration (saccades and fixations) response to visual cues during walking in PwPD and healthy controls (HC), and in PwPD who do and do not report FOG; and (2) investigate the relationship between visual exploration and gait response to visual cues in PwPD compared to HC, and in PwPD with and without FOG. We hypothesised that selective aspects of visual exploration will change with visual cues while walking, with those who report FOG experiencing the greatest improvement. Additionally, we hypothesised that changes in visual exploration will relate to gait improvement in PwPD, particularly within those that report FOG.

## Methods

### Participants

This interventional study examined visual exploration and gait response to visual cues during walking in PwPD that do and do not report FOG. Forty-three PD participants (n = 22 with self-reported FOG and n = 21 without FOG) and 17 age-matched HC participants at the Oregon Health and Science University (OHSU, Portland, Oregon, USA) Movement Disorders Clinic. Self-reported FOG was based upon question 1 of the new FOG Questionnaire after seeing the short clip related to the questionnaire. Subjects were categorized as “freezers” if they had experienced such a feeling or episode over the past month. All subjects provided their written informed consent prior to the experiment, and the study was approved by an Institutional Review Board (IRB) at OHSU, Portland, USA (#9903).

PwPD were included if they had clinical diagnosis of idiopathic PD by a movement disorder specialist according to UK brain bank criteria, a Hoehn and Yahr (H&Y) rating of stage II to III,^36^ aged ≥50 years, adequate vision and hearing (Snellen chart visual acuity ≥12/18), and able to walk and stand unaided. PD participants were excluded if they had; (1) cognitive impairment (ie, Montreal Cognitive Assessment [MoCA] score <21)^37^; (2) unstable medication for 1 month prior to study; (3) psychiatric co-morbidity; (4) acute lower back or lower/upper extremity pain; (5) peripheral neuropathy; (6) unable to comply with protocol; (7) rheumatic or orthopedic diseases affecting balance and gait.

### Data Collection

Data were collected at the Balance Disorders Laboratory, Oregon Health and Science University, Portland, Oregon, USA between July 2018 and November 2019. Each participant attended a 2-hour session at the laboratory. Participants were tested in their usual ON medication state (within 60 minutes of taking anti-Parkinsonian medications).

### Clinical Assessment

Participants underwent a series of demographic, clinical, and cognitive assessments. The following tests were administered; the Movement Disorder Society Unified Parkinson’s Disease Rating Scale Motor Subscale (MDS-UPDRS III),^38^ a 15-minute assessment of motor signs related to PD severity. Global cognition was assessed with the MoCA.^37^ Attention was measured with a computerized button pressing battery, involving simple reaction time (SRT), choice reaction time (CRT), and digit vigilance (DV).^39^ Executive function was measured using Royall’s clock drawing (CLOX 1&2).^40^ Working memory and visuo-spatial ability were measured through seated forward digit span and judgment of line orientation tasks,^41^ respectively. Basic visual functions of acuity and contrast sensitivity were assessed using standardized charts (logMar and logCS).

### Gait Assessment

Participants walked, at self-selected comfortable pace, over a 9 m straight path (with a 180° turn at each end) for 2 minutes. Four conditions were performed in a randomised order and included single and dual-task walking without, and with, visual cues. Under dual-task conditions, participants completed the forward digit span test whilst walking.

Initially, 2 numbers were played over a loudspeaker at a rate of 1 per second for the participant to recall and continued to a maximum of 9 numbers.^42^ For the visual cue condition, there were transverse black lines on the floor (at a distance 20% larger than participant baseline step length), which participants were asked to step over. Gait was measured using wearable inertial measurement units (128 Hz, Mobility Lab version 2, APDM, Inc.), which consisted of a gyroscope, accelerometer, and magnetometer. Inertial sensors were worn, on the wrists (x2), feet (x2), shanks (x2), sternum (x1), and lumbar spine (x1) attached via straps.

Gait outcomes were provided through the Mobility Lab proprietary software, and included stride length (m), gait speed (m/s), stride time variability, stride (s) time, and foot strike angle (°). These gait characteristics were chosen as they are valid^43^ and have previously been used in visual cueing studies.^24,44,45^ Only straight walking metrics were included. Turning was not examined, as the transverse nature of the visual cues along the 10 m pathway means that they are only used for straight walking (ie, there were no cues used specifically for the 180° turns).

### Visual Exploration

Mobile infrared eye-tracker (Tobii Pro Glasses 2, Tobii, Inc.) was used to objectively measure visual exploration during walking. Saccades (frequency, number, duration, amplitude) and fixations (duration) were measured during walking tasks. Raw eye-tracker data were analyzed with a validated custom-made Matlab algorithm^43^ with findings of this validation study showing good to excellent agreement Intraclass correlation coefficient (ICC > 0.75) across various gait features such as gait velocity, stride length, cadence and stride time in older adults, younger adults, and individuals with PD. The use of the mobile infrared eye tracker was previously validated by Stuart et al.^35^ and was found to be relatively robust, with it correctly detecting 85% of saccades made by participants during a walking task.

### Statistical Analysis

Statistical analysis was performed with IBM SPSS Statistics version 26. Normality of data was checked with Kolmogorov–Smirnov tests and visually assessed using box and scatterplots. Parametric statistics were used to assess descriptive demographic data (via Means, Standard Deviations, *T*-tests), and to examine visual exploration and gait changes in response to visual cueing. Separate linear mixed-effects models were conducted for each metric to examine the effect of intervention (cue, no-cue) and task (single, dual-task), with group (freezers, non-freezers) as a between-subjects factor. Further Pearson’s correlational analysis was conducted within each group (freezers, non-freezers, HC) to examine relationship between change scores (cue—no-cue) of visual exploration and gait. This was done for both single and dual task. Due to the exploratory nature of the study, significance level was set to *P* < .05 for statistical interpretation.

## Results

### Participants

Demographic, cognitive, visual, and clinical demographic data are displayed in Table 1. FOG, non-FOG, and HC were matched for age, height, weight, education, gender and across MoCA, CLOX1, CLOX2, and visual acuity scores. Non-freezers were more greatly impaired in terms of cognition, attention, executive function, working memory, visuo-spatial ability, and visual function than HC. This impairment was even greater in FOG in all domain’s bar the CLOX2 assessment as part of executive function. Freezers were significantly impaired when compared to HC in SRT (*P* = .022), CRT (*P* = .017), DV (*P* = .001), contrast sensitivity (*P* = .027), and Geriatric Depression Scale (GDS-15) (*P* = .007). Additionally, FOG had significantly more falls over the past 12 months (*P* = .031), and significantly worse attention (CRT [*P* = .029] and DV [*P* = .018]) compared to non-FOG.

**Table 1.** Demographic, Cognitive, Visual, and Clinical Characteristics.

Demographics	Parkinson’s disease (PD)	Healthy controls (HC)	Non-freezers	Freezing of gait (FOG)	*P*, all groups	*P*, HC vs non-freezers	*P*, HC vs FOG	*P*, non-freezers vs FOG
Age (years), mean (SD)	68.86 (6.56)	69.24 (7.97)	69.33 (4.97)	68.41 (7.87)	0.896	0.963	0.748	0.650
Sex	27M/16F	9M/8F	11M/10F	16M/6F	0.309	0.973	0.201	0.168
Height (cm)	172.76 (10.65)	170.33 (9.94)	169.91 (11.56)	175.49 (9.14)	0.400	0.735	0.186	0.190
Weight (kg)	81.89 (18.57)	75.24 (13.53)	76.87 (19.55)	86.68 (16.62)	0.962	0.772	0.854	0.919
GDS-15	6.09 (4.78)	3.24 (4.27)	4.96 (5.10)	7.18 (4.28)	**0.033**	0.275	**0.007**	0.128
Education (years)	16.68 (2.63)	17.82 (2.70)	17.14 (2.76)	16.59 (2.54)	0.365	0.450	0.152	0.499
Falls past 12 months	3.12 (8.65)	0.53 (1.18)	0.33 (0.58)	5.96 (11.54)	**0.018**	0.507	0.062	**0.031**
Handedness	41R/ 2L	14R/ 3L	21R/ 0L	20R/ 2L	0.145	**0.045**	0.428	0.157
Cognition								
MoCA	27.33 (2.50)	27.65 (1.69)	27.62 (2.22)	27.05 (2.77)	0.474	0.766	0.436	0.280
Executive function								
CLOX1	12.67 (1.63)	13.12 (1.45)	12.86 (1.68)	12.50 (1.60)	0.479	0.617	0.221	0.479
CLOX2	13.93 (1.03)	13.53 (1.46)	13.95 (1.24)	13.91 (0.81)	0.494	0.342	0.309	0.893
Visuo-spatial ability								
JLO	23.88 (6.73)	27.35 (2.45)	24.81 (4.68)	23.00 (8.26)	0.080	**0.050**	**0.043**	0.385
Working memory								
Digit span (sitting)	6.09 (1.17)	6 (1)	6.29 (1.23)	5.91 (1.11)	0.529	0.445	0.793	0.297
Attention								
SRT	374.96 (78.89)	343.35 (44.90)	351.12 (66.18)	397.72 (84.66)	**0.030**	0.682	**0.022**	0.052
CRT	569.84 (148.95)	506.78 (60.75)	519.59 (97.50)	617.81 (174.30)	**0.011**	0.64	**0.017**	**0.029**
DV	521.08 (83.51)	464.71 (41.98)	490.72 (66.86)	550.07 (88.81)	**0.001**	0.172	**0.001**	0.018
Visual function								
Visual acuity binocular	0.07 (0.15)	0.04 (0.13)	0.06 (0.18)	0.08 (0.11)	0.692	0.725	0.309	0.642
Contrast sensitivity	1.57 (0.17)	1.66 (0.13)	1.57 (0.20)	1.56 (0.14)	0.127	0.118	**0.027**	0.855
Clinical								
Disease duration	7.77 (5.73)	n/a	5.50 (2.95)	9.93 (6.88)	—	—	—	**0.010**
H & Y stage	I (0), II (39), III(4)	n/a	I (0), II (21), III (0)	I (0), II (18), III (4)	—	—	—	**0.041**
LEDD	817.46 (409.61)	n/a	628.64 (373.81)	997.69 (364.42)	—	—	—	**0.020**
UPDRS-III	31.88 (14.34)	n/a	25.38 (12.34)	38.09 (13.56)	—	—	—	**0.003**
FOG-Q	7.74 (9.42)	n/a	0.00 (0.00)	15.14 (7.75)				**<0.001**
Dual-task cognitive								
Walk (% error)	31.91 (19.76)	21.76 (20.40)	30.99 (19.61)	32.80 (20.33)	0.081	0.165	0.102	0.769
Visual cue (% error)	32.07 (27.52)	27.88 (20.97)	32.99 (27.89)	31.19 (27.79)	0.574	0.536	0.685	0.834

Abbreviations: SD, standard deviations; cm, centimetres; kg, kilogram; MoCA, Montreal Cognitive Assessment; JLO, judgment of line orientation; SRT, simple reaction time; CRT, cued reaction time; DV, digit vigilance; H & Y, Hoehn and Yahr; LEDD, levodopa equivalent daily dose; UPDRS-III, Unified Parkinson’s Disease Rating Scale-III; FOG-Q, freezing of gait questionnaire; M, male; F, female.

Significance level *P* < .05 highlighted in bold.

### Eye Movement Outcomes: Effect of Visual Cues

In general, there were no significant differences in saccadic activity between the groups (Table 2) and dual tasking was found to have no significant effect on the eye movement outcomes (*P* < .01, Table 2). However, Table 2 shows that visual cues significantly increased saccade frequency (*P* ≤ .01), but reduced saccade peak velocity and amplitude in all groups (HC, freezers, non-freezers, *P* ≤ .01). Additionally, a cue by group interaction showed significant reduction in saccade peak velocity (*P* *=* .03) and increased saccade duration (*P* *=* .05) with visual cues in HC and non-freezers compared to freezers (Table 2). Post hoc analysis also showed specific reduction in saccade peak velocity in non-freezers with visual cues compared to without visual cues (*P* *=* .006), whereas HC and freezers did not significantly reduce their saccade peak velocity.

**Table 2.** Linear Mixed Effects Model Results for Gait and Eye Tracking Variables Across All Participants Groups With and Without Visual Cues and During Single and Dual Tasks.

	Single task				Single task—visual cues				Dual task				Dual task—visual cues				Linear mixed effect model			
	PwPD	HC	FOG	Non-FOG	PwPD	HC	FOG	Non-FOG	PwPD	HC	FOG	Non-FOG	PwPD	HC	FOG	Non-FOG	Task (ST,, DT) *P*	Cue (cue, no cue) *P*	Group (HC, FOG, non-FOG) *P*	Cue × group *P*
Gait																				
Stride length (m)	1.05 (0.16)	1.19 (0.09)	0.98 (0.15)	1.12 (0.14)	1.21 (0.17)	1.35 (0.11)	1.13 (0.17)	1.29 (0.14)	1.00 (0.16)	1.15 (0.10)	0.94 (0.17)	1.07 (0.12)	1.20 (0.19)	1.34 (0.12)	1.11 (0.21)	1.28 (0.14)	**<.01**	**<.01**	.20	.89
Gait speed (m/s)	0.98 (0.17)	1.12 (0.14)	0.89 (0.16)	1.07 (0.14)	1.01 (0.20)	1.20 (0.15)	0.93 (0.19)	1.11 (0.17)	0.92 (0.18)	1.07 (0.15)	0.84 (0.19)	1.01 (0.13)	0.97 (0.21)	1.16 (0.15)	0.89 (0.23)	1.06 (1.56)	**<.01**	.37	.70	**<.01**
Stride time variability (s)	0.04 (0.02)	0.03 (0.01)	0.04 (0.02)	0.03 (0.01)	0.06 (0.03)	0.04 (0.01)	0.07 (0.03)	0.05 (0.02)	0.04 (0.02)	0.03 (0.01)	0.04 (0.19)	0.03 (0.01)	0.06 (0.03)	1.18 (0.11)	0.06 (0.03)	0.06 (0.04)	.20	**<.01**	.75	.10
Stride time (s)	1.08 (0.10)	1.07 (0.09)	1.11 (0.10)	1.06 (0.10)	1.22 (0.15)	1.14 (0.09)	1.25 (0.15)	1.19 (0.14)	1.10 (0.10)	1.09 (0.10)	1.13 (0.10)	1.08 (0.10)	1.26 (0.16)	1.18 (0.11)	1.29 (0.18)	1.23 (0.15)	**<.01**	**<.01**	.15	**<.01**
Foot strike angle (°)	12.38 (5.57)	18.03 (2.93)	9.66 (5.39)	15.23 (4.24)	17.12 (5.22)	22.29 (2.60)	15.25 (5.05)	19.08 (4.75)	11.45 (5.51)	17.21 (2.80)	8.97 (5.60)	14.05 (4.12)	17.0 (5.46)	22.79 (2.94)	14.74 (5.78)	19.26 (4.11)	**.04**	**<.01**	.12	.51
Eye movement																				
Saccade frequency (Hz)	2.49 (0.84)	2.40 (0.83)	2.61 (0.86)	2.37 (0.81)	3.25 (0.56)	2.77 (0.62)	3.28 (0.65)	3.21 (0.45)	2.57 (0.74)	2.49 (0.80)	2.52 (0.69)	2.63 (0.80)	3.21 (0.57)	2.92 (0.58)	3.33 (0.53)	3.07 (0.60)	.30	**<.01**	.51	.13
Saccade duration (s)	0.07 (0.02)	0.06 (0.01)	0.07 (0.02)	0.07 (0.02)	0.07 (0.02)	0.06 (0.02)	0.08 (0.03)	0.07 (0.02)	0.07 (0.02)	0.06 (0.01)	0.07 (0.02)	0.07 (0.02)	0.07 (0.02)	0.07 (0.02)	0.08 (0.02)	0.06 (0.02)	.79	.19	.07	**.05**
Saccade peak velocity (°/s)	588.44 (69.47)	563.08 (39.55)	593.72 (70.82)	583.16 (69.51)	560.37 (81.90)	548.37 (66.98)	574.84 (93.65)	544.29 (65.40)	599.21 (64.77)	567.99 (36.69)	604.00 (55.27)	594.19 (74.60)	570.05 (87.51)	558.58 (57.50)	592.33 (89.33)	544.05 (79.99)	.10	**<.01**	.08	**.03**
Saccade amplitude (°)	10.67 (0.89)	10.76 (0.78)	10.67 (0.82)	10.68 (0.97)	9.89 (0.78)	10.10 (1.18)	9.89 (0,85)	9.89 (0.72)	11.00 (1.10)	10.65 (0.96)	11.26 (1.28)	10.72 (0.83)	10.15 (1.02)	10.03 (0.89)	10.19 (1.06)	10.12 (1.00)	.11	**<.01**	.90	.83
Fixation duration (m/s)	169.28 (36.03)	184.52 (45.83)	166.55 (34.44)	171.86 (38.19)	186.22 (90.79)	270.76 (122.16)	174.55 (49.76)	198.53 (120.50)	179.34 (35.46)	186.34 (51.36)	173.70 (27.19)	185.27 (42.44)	182.71 (72.30)	216.61 (137.66)	174.38 (47.88)	191.49 (92.10)	.57	.60	.42	.09

Abbreviations: PwPD, people with PD; non-FOG, non-freezers; FOG, freezers; HC, healthy controls; ST, single task; DT, dual task; m, meters; m/s, meters per second; s, seconds; °, degrees; °/s, degrees per second; Hz, hertz.

*P* = Significance level (*P* < .05, highlighted in bold). Means given for each eye movement and gait outcome and standard deviations indicated in brackets.

### Gait Outcomes: Effect of Visual Cues

PwPD generally had worse gait than HC, and dual tasking worsened all gait variables apart from stride time variability (all *P* ≤ .05). Visual cues improved stride length, foot strike angle, stride time, and stride time variability in all groups for both single and dual task (*P* ≤ .01). A cue by group interaction showed that stride time increased significantly with visual cues for all 3 subgroups (post hoc analysis: freezers, non-freezers, and HC; *P* ≤ .001, *P* ≤ .001, *P* *=* .003 respectively), but freezers had the greatest increase. Post hoc analysis did not detect a significant intragroup change in gait speed across walking conditions.

Visual cues did not influence cognitive dual-task outcomes. Specifically, there was no significant difference in dual-task cognitive task percentage error score for any of the groups during walking without or with the visual cues (Table 1). Additionally, there was not a significant difference between dual-task cognitive task error score from walking without and with visual cues for HC (*P* = .077), PD (*P* = .966); non-freezers (*P* = .715) or freezers (*P* = .744).

### Association Between Cueing-Related Changes in Visual Exploration and Gait

Significant relationships between cue-related changes in visual exploration and gait with visual cues were observed for PwPD under both single and dual-task conditions, but not for HC (Tables 3 and 4). Specifically, under single-task conditions, greater stride length related to less change in saccade duration (*r* = −.5, *P* *=* .042), foot strike time related to less change in saccade amplitude (*r* = −.5, *P* *=* .033), and stride time variability related to less change in saccade velocity and saccade amplitude (*r* = −.5, *P* *=* .040; *r* = −.6, *P* = .008 respectively) with visual cues in non-freezers. Additionally, more frequent saccades with visual cues related to increased stride length in freezers (*r* = .5, *P* *=* .040) with visual cues under single task. Under dual-task conditions, increased speed with visual cues related to greater saccade frequency (*r* = −.7, *P* = .002) in non-freezers. In freezers, increased stride length and foot strike angle related to increased saccade frequency (*r* = .7, *P* = .002; *r* = .5, *P* = .024 respectively) with visual cues. Furthermore, increased stride length and foot strike angle related to increased saccade duration with visual cues in freezers (*r* = .5, *P* = .017; *r* = .5, *P* = .034). Overall, this evidence suggests that there are PD and freezer specific associations between cue-related changes in visual exploration and cue-related changes in gait.

**Table 3.** Relationships Between Cueing-Related Changes in Visual Exploration and Gait in Single Task.

	Δ Speed	Δ Stride length	Δ Foot strike angle	Δ Foot strike time	Δ Stride time variability
Non-FOG					
Δ Saccade frequency change	.049 (.851)	−.111 (.671)	.067 (.797)	−.190 (.466)	−.028 (.914)
Δ Saccade duration	−.285 (.268)	−**.498 (.042)**	−.402 (.110)	−.204 (.433)	−.164 (.528)
Δ Saccade velocity	−.023 (.930)	−.433 (.083)	−.202 (.438)	−.430 (.085)	−**.501 (.040)**
Δ Saccade amplitude	.366 (.148)	−.141 (.589)	−.129 (.621)	−**.518 (.033)**	−**.616 (.008)**
Δ Fixation duration	.344 (.177)	.275 (.285)	.237 (.361)	−.079 (.763)	−.096 (.713)
FOG					
Δ Saccade frequency	.249 (.320)	**.489 (.040)**	.325 (.188)	−.020 (.938)	**.517 (.028)**
Δ Saccade duration	.093 (.715)	.385 (.115)	.411 (.090)	.281 (.258)	−.276 (.268)
Δ Saccade velocity change	.127 (.614)	.146 (.564)	.223 (.373)	.173 (.493)	−.076 (.763)
Δ Saccade amplitude	.073 (.773)	−.223 (.374)	−.109 (.940)	.019 (.940)	.279 (.263)
Δ Fixation duration	−.048 (.855)	−.190 (.465)	.218 (.401)	.041 (.877)	.028 (.915)
HC					
Δ Saccade frequency	−.045 (.868)	.101 (.709)	−.181 (.503)	.266 (.319)	.110 (.684)
Δ Saccade duration	.280 (.294)	.367 (.162)	−.240 (.370)	.252 (.347)	.000 (1.00)
Δ Saccade velocity	.059 (.828)	.204 (.448)	−.129 (.634)	.300 (.259)	−.027 (.921)
Δ Saccade amplitude	−.091 (.738)	.029 (.915)	.093 (.732)	.164 (.544)	.053 (.845)
Δ Fixation duration	−.284 (.286)	−.207 (.443)	−.266 (.139)	.073 (.788)	−.094 (.728)

Significance level *P* < .05 highlighted in bold. *P* values highlighted in brackets.

Abbreviations: Δ, change; non-FOG, non-freezers; FOG, freezers; HC, healthy controls.

**Table 4.** Relationships Between Cueing-Related Changes in Visual Exploration and Gait in Dual Task.

	Δ Speed	Δ Stride length	Δ Foot strike angle	Δ Foot strike time	Δ Stride time variability
Non-FOG					
Δ Saccade frequency	−**.687 (.002)**	−.375 (.138)	−.429 (.086)	.334 (.189)	−.027 (.919)
Δ Saccade duration	−.436 (.080)	−.297 (.247)	−.281 (.275)	.133 (.612)	−.224 (.386)
Δ Saccade velocity	−.341 (.180)	−.360 (.156)	−.417 (.096)	−.007 (979)	−.035 (.895)
Δ Saccade amplitude	.030 (.910)	−.255 (.323)	−.284 (.269)	−.282 (.274)	.097 (.710)
Δ Fixation duration	.297 (.263)	**.610 (.012)**	**.748 (<.001)**	.293 (.272)	.195 (.469)
FOG					
Δ Saccade frequency	.419 (.074)	**.665 (.002)**	**.539 (.017)**	−.099 (.688)	.430 (.066)
Δ Saccade duration	.335 (.161)	**.515 (.024)**	**.487 (.034)**	.084 (.733)	.192 (.430)
Δ Saccade velocity change	.167 (.485)	.297 (.217)	.223 (.358)	.174 (.475)	−.017 (.943)
Δ Saccade amplitude	−.071 (.772)	−.055 (.823)	−.214 (.379)	.195 (.425)	−.125 (.609)
Δ Fixation duration	−.132 (.602)	.125 (.602)	.294 (.236)	.496 (.036)	−.024 (.926)
HC					
Δ Saccade frequency	.319 (.246)	−.151 (.591)	−.017 (.951)	.199 (.476)	−.265 (.339)
Δ Saccade duration	.166 (.553)	−.112 (.691)	−.420 (.119)	−.235 (.400)	−.131 (.641)
Δ Saccade velocity	.160 (.568)	−.192 (.494)	−.406 (.133)	−.297 (.282)	−.016 (.956)
Δ Saccade amplitude	.060 (.833)	.011 (.969)	.217 (.438)	.009 (.975)	−.033 (.908)
Δ Fixation duration	.059 (.834)	−.058 (.838)	.004 (.989)	−.095 (.735)	.261 (.348)

Significance level *P* < .05 highlighted in bold. *P* values highlighted in brackets.

Abbreviations: Δ, change; non-FOG, non-freezers; FOG, freezers; HC, healthy controls.

## Discussion

To our knowledge, this is the first study to comprehensively explore the influence of visual cues on visual exploration (saccade and fixation outcomes) during gait in healthy older adults and PwPD who do and do not report FOG. Unsurprisingly, visual cues produced significant improvements in gait compared to usual walking across all groups. In line with our hypothesis, visual cueing significantly influenced selective visual exploration outcomes compared to usual walking with increased saccade frequency, decreased saccade peak velocity and amplitude observed across all groups, which demonstrates that visuo-cognitive processes underpin cue response regardless of group. However, in contrast to our hypothesis, saccadic peak velocity had significant reduction with visual cues in non-freezers compared to HC and those with freezing, which highlights that selective visuo-cognitive processes may response differently in non-freezers compared to other groups. Additionally, in line with our hypothesis, changes in visual exploration outcomes related to gait improvements with cueing in PwPD, but in contrast to our hypothesis relationships were selective across freezers and non-freezers. Our results highlight that visuo-cognitive processes underpin visual cue response in PwPD, with processes being subtly different across those who do and do not self-report FOG.

### Visual Exploration When Walking: Response to Visual Cues

This study demonstrates that visual cues were found to significantly increase saccade frequency during gait across all groups, which is in keeping with the findings of Stuart et al.^25^ and Vitorio et al.^19^ where visuo-cognitive, particularly attentional, mechanisms were reported to underpin cue response. Similarly, Horowitz et al.^46^ also found that highly visible targets and specific task goals resulted in improved saccadic activity in PwPD during static tasks. The current study went beyond previous work by recording comprehensive visual exploration with high resolution (>100 Hz) mobile infra-red eye-tracking, allowing additional measures of velocity, duration, and amplitude of eye movements to be measured. Previous research has been unable to provide comprehensive visual exploration outcomes, as prior studies have lacked a validated algorithm to obtain outcomes from eye-tracking data, and they also had lower sampling frequency (30-50 Hz) eye-tracking devices.^14,19,25^ It is known that frequencies ≥100 Hz are required to accurately attain comprehensive visual exploration outcomes (saccades and fixations),^35^ which has limited previous studies due to the technological limitations and limits direct comparison to this study.

Visual cues significantly reduced saccade peak velocity and amplitude in all groups (HC, freezers, non-freezers), which may relate to enhanced goal directed visuo-cognitive processing. Reflexive saccades (triggered via visual distraction) typically have greater velocity that voluntary attentionally controlled saccades,^47^ therefore the reduction in saccade velocity and the narrower amplitude of eye movements suggests more voluntary saccades were elicited with visual cues (ie, focus on the visual cues and avoidance of distractors).^28^ Interestingly, saccade peak velocity was particularly reduced in non-freezers, with HC and freezers responding in a similar manner, which may indicate that visuo-cognitive processes underpinning cue response are similar in HC and freezers. Visuo-cognitive processes, particularly attentional processing, has been implicated in visual cue response in PwPD in our previous research.^23,25,26,28,48^ To examine the influence of attention, the present study used attentional distraction through dual tasking, which was found to have no significant effect on any of the eye movement outcomes when walking with visual cues compared to single-task conditions, which is similar to findings by Stuart et al.^25^ Visual cues may reduce the visuo-cognitive processing of the environment that is required when walking in PwPD which allows specific focus on task relevant areas of interest (lines on the floor) and frees attentional resources to be applied to concurrent tasks (ie, the dual-task).^49^

### Gait Outcomes When Walking: Response to Visual Cues

PwPD (freezers and non-freezers) were found to have significant impairment in gait speed, stride length, and foot strike angle compared to HC. These gait impairments amongst PwPD are already well established having been highlighted previous research.^50-52^ Additionally, dual-tasking made these gait variables worse across all groups, particularly stride time, in line with previous literature.^53^ As expected, visual cues increased stride length, foot strike angle, and stride time in all groups, similar to previous research in PwPD.^17,18,20^ Slower stride time with visual cues occurred across all groups (HC, freezer, non-freezer) but was particularly slow in freezers, which may indicate a more cautious gait approach with cues.

Our findings show significant relationship between gait outcomes and visual exploration outcomes in PwPD but not HC, which highlights the increased visuo-cognitive processing required for visual cue response in PwPD. More specifically, relationships existed between gait and visual exploration changes with visual cues for freezers and non-freezers. Results highlighted that improved gait (increased speed, stride length, etc.) was related to improved visual exploration (more frequent saccades, etc.) with visual cues, but these were selective to group (freezer vs non-freezer) and task (single vs dual). This is an important finding as it shows that underlying visuo-cognitive mechanisms may be different in Parkinson’s compared to controls and within freezer versus non-freezer sub-groups.^48,54^

### Limitations

There are several limitations to highlight. First, this study only examined immediate effect of visual cues on visual exploration and gait outcomes, rather than continuous home-based cue use as performed in clinical practice. This gives no indication of whether any improvements in these outcomes from visual cues are lasting or whether they continue to occur in the absence of visual cues. Secondly, this study only examined information collected about eye movements and does not investigate how this information is processed through brain activity, or how freezers and non-freezers may process information differently. This could potentially be explored in further research and contribute to our understanding of the mechanisms behind visual cue response (ie, mobile brain imaging studies^26,55-57^) and aid the development of therapeutic interventions. Finally, there is a lack of contextual analysis whereby there is no means of determining where or what people look at whilst walking. This could, for example, be used to determine how far ahead individuals look while walking and a validated algorithm has been presented in previous work to semi-automate this.^27^ Contextual analysis would be useful in future investigation as PwPD have been shown to visually explore their environments with less efficiency than HC.^28^

## Conclusion

This study provides valuable insight into the visual cue response of visual exploration during walking in HC and PwPD, who do and do not display FOG, which may improve mobility and reduce falls risk. Overall, visual cues significantly influenced several visual exploration and gait outcomes in HC and Parkinson’s participants, which highlights the requirement for visuo-cognitive processing to respond to cues in PwPD. Additionally, there were freezer and non-freezer specific associations between cueing-related changes in visual exploration and gait that suggests different visuo-cognitive mechanisms may underpin cue response in these 2 sub-groups.

## References

[1] Sawada M Wada-Isoe K Hanajima R Nakashima K. Clinical features of freezing of gait in Parkinson’s disease patients. Brain Behav. 2019;9(4):e01244. doi:10.1002/brb3.1244 PMC6456785 30851088

[2] Moore ST MacDougall HG Ondo WG. Ambulatory monitoring of freezing of gait in Parkinson’s disease. J Neurosci Methods. 2008;167(2):340-348. doi:10.1016/j.jneumeth.2007.08.023 17928063

[3] Heremans E Nieuwboer A Vercruysse S. Freezing of gait in Parkinson’s disease: where are we now? Current neurology and neuroscience reports. 2013;13(6):350. 23625316 10.1007/s11910-013-0350-7

[4] Ge H-L Chen X-Y Lin Y-X , et al. The prevalence of freezing of gait in Parkinson’s disease and in patients with different disease durations and severities. Chin Neurosurg J. 2020;6(1):17. doi:10.1186/s41016-020-00197-y 32922946 PMC7398304

[5] Zhang W-S Gao C Tan Y-Y Chen S-D. Prevalence of freezing of gait in Parkinson’s disease: a systematic review and meta-analysis. J Neurol. 2021;268(11):4138-4150. doi:10.1007/s00415-021-10685-5 34236501

[6] Kalia LV Lang AE. Evolving basic, pathological and clinical concepts in PD. Nat Rev Neurol. 2016;12(2):65-66. doi:10.1038/nrneurol.2015.249 26782330

[7] Allen NE Schwarzel AK Canning CG. Recurrent falls in Parkinson’s disease: a systematic review. Parkinson’s Dis. 2013;2013:906274. doi:10.1155/2013/906274 23533953 PMC3606768

[8] Schrag A Choudhury M Kaski D Gallagher DA. Why do patients with Parkinson’s disease fall? A cross-sectional analysis of possible causes of falls. NPJ Parkinson’s Dis. 2015;1(1):15011. doi:10.1038/npjparkd.2015.11 28409181 PMC5388183

[9] Zhang L-L Canning SD Wang X-P. Freezing of gait in parkinsonism and its potential drug treatment. Curr Neuropharmacol. 2016;14(4):302-306. doi:10.2174/1570159x14666151201190040 26635194 PMC4876585

[10] Gao C Liu J Tan Y Chen S. Freezing of gait in Parkinson’s disease: pathophysiology, risk factors and treatments. Transl Neurodegener. 2020;9(1):12. doi:10.1186/s40035-020-00191-5 32322387 PMC7161193

[11] Nonnekes J Snijders AH Nutt JG Deuschl G Giladi N Bloem BR. Freezing of gait: a practical approach to management. Lancet Neurol. 2015;14(7):768-778. doi:10.1016/S1474-4422(15)00041-1 26018593

[12] Morris ME Taylor NF Watts JJ , et al. A home program of strength training, movement strategy training and education did not prevent falls in people with Parkinson’s disease: a randomised trial. J Physiother. 2017;63(2):94-100. doi:10.1016/j.jphys.2017.02.015 28342682

[13] Azulay J-P Mesure S Blin O. Influence of visual cues on gait in Parkinson’s disease: contribution to attention or sensory dependence? J Neurol Sci. 2006;248(1):192-195. doi:10.1016/j.jns.2006.05.008 16765379

[14] Beck EN Ehgoetz Martens KA Almeida QJ. Freezing of gait in Parkinson’s disease: an overload problem? PLoS One. 2015;10(12):e0144986. doi:10.1371/journal.pone.0144986 PMC4682987 26678262

[15] Ehgoetz Martens KA Pieruccini-Faria F Almeida QJ. Could sensory mechanisms be a core factor that underlies freezing of gait in Parkinson’s disease? PLoS One. 2013;8(5):e62602. doi:10.1371/journal.pone.0062602 PMC3648560 23667499

[16] Nieuwboer A Kwakkel G Rochester L , et al. Cueing training in the home improves gait-related mobility in Parkinson’s disease: the RESCUE trial. J Neurol Neurosurg Psychiatry. 2007;78(2):134. doi:10.1136/jnnp.200X.097923 17229744 PMC2077658

[17] Lee SJ Yoo JY Ryu JS Park HK Chung SJ. The effects of visual and auditory cues on freezing of gait in patients with Parkinson disease. Am J Phys Med Rehabil. 2012;91(1):2-11. 22157432 10.1097/PHM.0b013e31823c7507

[18] Suteerawattananon M Morris GS Etnyre BR Jankovic J Protas EJ. Effects of visual and auditory cues on gait in individuals with Parkinson’s disease. J Neurol Sci. 2004;219(1):63-69. doi:10.1016/j.jns.2003.12.007 15050439

[19] Vitório R Lirani-Silva E Pieruccini-Faria F Moraes R Gobbi LTB Almeida QJ. Visual cues and gait improvement in Parkinson’s disease: which piece of information is really important? Neuroscience. 2014;277:273-280. doi:10.1016/j.neuroscience.2014.07.024 25065625

[20] Jiang Y Norman KE. Effects of visual and auditory cues on gait initiation in people with Parkinson’s disease. Clin Rehabil. 2006;20(1):36-45. doi:10.1191/0269215506cr925oa 16502748

[21] Peterson DS Smulders K. Cues and attention in Parkinsonian gait: potential mechanisms and future directions. Front Neurol. 2015;6:255. doi:10.3389/fneur.2015.00255 26696955 PMC4672041

[22] Morris ME Martin CL Schenkman ML. Striding out with Parkinson disease: evidence-based physical therapy for gait disorders. Phys Ther. 2010;90(2):280-288. doi:10.2522/ptj.20090091 20022998 PMC2816030

[23] Stuart S Alcock L Hunt D Galna B Lord S Rochester L. Visual Exploration During Gait in Parkinson’s Disease: Response to VISUAL cues. Newcastle University; 2017.

[24] Spaulding SJ Barber B Colby M Cormack B Mick T Jenkins ME. Cueing and gait improvement among people with Parkinson’s disease: a meta-analysis. Arch Phys Med Rehabil. 2013;94(3):562-570. doi:10.1016/j.apmr.2012.10.026 23127307

[25] Stuart S Lord S Galna B Rochester L. Saccade frequency response to visual cues during gait in Parkinson’s disease: the selective role of attention. Eur J Neurosci. 2018;47(7):769-778. doi:10.1111/ejn.13864 29431890

[26] Stuart S Wagner J Makeig S Mancini M. Brain activity response to visual cues for gait impairment in Parkinson’s disease: an EEG study. Neurorehabil Neural Repair. 2021;35(11):996-1009. doi:10.1177/15459683211041317 34505536 PMC8593320

[27] Stuart S Hunt D Nell J , et al. Do you see what I see? Mobile eye-tracker contextual analysis and inter-rater reliability. Med Biol Eng Comput. 2018;56(2):289-296. doi:10.1007/s11517-017-1669-z 28712014 PMC5790862

[28] Hunt D Stuart S Nell J , et al. Do people with Parkinson’s disease look at task relevant stimuli when walking? An exploration of eye movements. Behav Brain Res. 2018;348:82-89. doi:10.1016/j.bbr.2018.03.003 29559336

[29] Stuart S Lord S Hill E Rochester L. Gait in Parkinson’s disease: a visuo-cognitive challenge. Neurosci Biobehav Rev. 2016;62:76-88. doi:10.1016/j.neubiorev.2016.01.002 26773722

[30] Kimmig H Greenlee M Gondan M Schira M Kassubek J Mergner T. Relationship between saccadic eye movements and cortical activity as measured by fMRI: quantitative and qualitative aspects. Exp Brain Res. 2001;141(2):184-194. doi:10.1007/s002210100844 11713630

[31] Galna B Lord S Daud D Archibald N Burn D Rochester L. Visual sampling during walking in people with Parkinson’s disease and the influence of environment and dual-task. Brain Res. 2012;1473:35-43. doi:10.1016/j.brainres.2012.07.017 22824332

[32] Stuart S Galna B Delicato LS Lord S Rochester L. Direct and indirect effects of attention and visual function on gait impairment in Parkinson’s disease: influence of task and turning. Eur J Neurosci. 2017;46(1):1703-1716. doi:10.1111/ejn.13589 28444834

[33] Salvucci DD Goldberg JH. Identifying fixations and saccades in eye-tracking protocols. Paper presented at: Proceedings of the 2000 Symposium on Eye Tracking Research & Applications, Palm Beach Gardens, FL, USA, 2000.

[34] Nemanich ST Earhart GM. Freezing of gait is associated with increased saccade latency and variability in Parkinson’s disease. Clin Neurophysiol. 2016;127(6):2394-2401. doi:10.1016/j.clinph.2016.03.017 27178858 PMC4867191

[35] Stuart S Galna B Lord S Rochester L Godfrey A . Quantifying saccades while walking: validity of a novel velocity-based algorithm for mobile eye tracking. 2014:5739-5742. 10.1109/EMBC.2014.6944931 25571299

[36] Hoehn MM Yahr MD. Parkinsonism: onset, progression, and mortality. Neurology. 1998;50(2):318. 9484345 10.1212/wnl.50.2.318

[37] Dalrymple-Alford JC MacAskill MR Nakas CT , et al. The MoCA. Neurology. 2010;75(19):1717. doi:10.1212/WNL.0b013e3181fc29c9 21060094

[38] Goetz CG Fahn S Martinez-Martin P , et al. Movement Disorder Society-sponsored revision of the Unified Parkinson’s Disease Rating Scale (MDS-UPDRS): process, format, and clinimetric testing plan. Movement disorders. 2007;22(1):41-47. 17115387 10.1002/mds.21198

[39] Corani G Edgar C Marshall I Wesnes K Zaffalon M . Classification of Dementia Types From Cognitive Profiles Data. Springer Berlin Heidelberg; 2006:470-477.

[40] Royall DR Cordes JA Polk M. CLOX: an executive clock drawing task. J Neurol Neurosurg Psychiatry. 1998;64(5):588. doi:10.1136/jnnp.64.5.588 9598672 PMC2170069

[41] Wechsler D. A standardized memory scale for clinical use. J Psychol. 1945;19(1):87-95. doi:10.1080/00223980.1945.9917223

[42] Wilde NJ Strauss E Tulsky DS. Memory span on the Wechsler Scales. J Clin Exp Neuropsychol. 2004;26(4):539-549. doi:10.1080/13803390490496605 15512941

[43] Morris R Stuart S McBarron G Fino PC Mancini M Curtze C. Validity of Mobility Lab (version 2) for gait assessment in young adults, older adults and Parkinson’s disease. Physiol Meas. 2019;40(9):095003. 31470423 10.1088/1361-6579/ab4023 PMC8072263

[44] De Icco R Tassorelli C Berra E Bolla M Pacchetti C Sandrini G . Acute and chronic effect of acoustic and visual cues on gait training in Parkinson’s disease: a randomized, controlled study. Parkinson’s Dis. 2015;2015:978590. doi:10.1155/2015/978590 26693384 PMC4674608

[45] Lewis GN Byblow WD Walt SE. Stride length regulation in Parkinson’s disease: the use of extrinsic, visual cues. Brain. 2000;123(10):2077-2090. doi:10.1093/brain/123.10.2077 11004125

[46] Horowitz TS Choi WY Horvitz JC Côté LJ Mangels JA. Visual search deficits in Parkinson’s disease are attenuated by bottom-up target salience and top-down information. Neuropsychologia. 2006;44(10):1962-1977. doi:10.1016/j.neuropsychologia.2006.01.037 16580700

[47] Xu-Wilson M Zee DS Shadmehr R. The intrinsic value of visual information affects saccade velocities. Exp Brain Res 2009;196(4):475-481. doi:10.1007/s00221-009-1879-1 19526358 PMC2771693

[48] Das J Vitorio R Butterfield A , et al. Visual cues for turning in Parkinson’s disease. Sensors. 2022;22(18):6746. doi:10.3390/s22186746 36146096 PMC9502260

[49] Chan F Armstrong IT Pari G Riopelle RJ Munoz DP. Deficits in saccadic eye-movement control in Parkinson’s disease. Neuropsychologia. 2005;43(5):784-796. doi:10.1016/j.neuropsychologia.2004.06.026 15721191

[50] Ginis P Pirani R Basaia S , et al. Focusing on heel strike improves toe clearance in people with Parkinson’s disease: an observational pilot study. Physiotherapy. 2017;103(4):485-490. doi:10.1016/j.physio.2017.05.001 28784427

[51] Henmi O Shiba Y Saito T , et al. Spectral analysis of gait variability of stride interval time series: comparison of young, elderly and Parkinson’s disease patients. J Phys Ther Sci. 2009;21(2):105-111. doi:10.1589/jpts.21.105

[52] Baltadjieva R Giladi N Gruendlinger L Peretz C Hausdorff JM. Marked alterations in the gait timing and rhythmicity of patients with de novo Parkinson’s disease. Eur J Neurosci. 2006;24(6):1815-1820. doi:10.1111/j.1460-9568.2006.05033.x 17004944

[53] Brauer SG Woollacott MH Lamont R , et al. Single and dual task gait training in people with Parkinson’s disease: a protocol for a randomised controlled trial. BMC Neurol. 2011;11(1):90. doi:10.1186/1471-2377-11-90 21791117 PMC3155494

[54] Fasano A Canning CG Hausdorff JM Lord S Rochester L. Falls in Parkinson’s disease: a complex and evolving picture. Mov Disord. 2017;32(11):1524-1536. doi:10.1002/mds.27195 29067726

[55] Belluscio V Stuart S Bergamini E Vannozzi G Mancini M. The association between prefrontal cortex activity and turning behavior in people with and without freezing of gait. Neuroscience. 2019;416:168-176. doi:10.1016/j.neuroscience.2019.07.024 31330231 PMC7778469

[56] Vitorio R Stuart S Giritharan A Quinn J Nutt JG Mancini M. Changes in prefrontal cortical activity and turning in response to dopaminergic and cholinergic therapy in Parkinson’s disease: a randomized cross-over trial. Parkinsonism Relat Disord. 2021;86:10-14. doi:10.1016/j.parkreldis.2021.03.014 33813359

[57] Vitorio R Stuart S Mancini M. Executive control of walking in people with Parkinson’s disease with freezing of gait. Neurorehabil Neural Repair. 2020;34(12):1138-1149. doi:10.1177/1545968320969940 33155506 PMC7704905

